# Two surgical strategies (early carotid reperfusion vs. Central aortic repair-first) of acute type a aortic dissection complicated with cerebral malperfusion syndrome: a meta-analysis and systematic review

**DOI:** 10.1186/s12872-024-03910-2

**Published:** 2024-05-07

**Authors:** Kang He, Xiaoli Qin, Mei Li, Longrong Bian, Honghua Yue, Weitao Liang, Zhong Wu

**Affiliations:** Department of Cardiovascular surgery, West China Hospital of Sichuan University, Chengdu, Sichuan 610041 China

**Keywords:** Acute type a aortic dissection, Cerebral malperfusion, Central repair, Early reperfusion

## Abstract

**Objective:**

Cerebral malperfusion (CM) is a common comorbidity in acute type A aortic dissection (ATAAD), which is associated with high mortality and poor neurological prognosis. This meta-analysis investigated the surgical strategy of ATAAD patients with CM, aiming to compare the difference in therapeutic effectiveness between the central repair-first and the early reperfusion-first according to clinical outcomes.

**Methods:**

The meta-analysis and systematic review was conducted based on studies sourced from the PubMed, Embase, and Cochrane literature database, in which cases of ATAAD with CM underwent surgical repair were included. Data for baseline characteristics, mortality, survival were extracted, and risk ratio (RR) values and the pooled mortality were calculated.

**Results:**

A total of 17 retrospective studies were analyzed, including 1010 cases of ATAAD with CM underwent surgical repair. The pooled early mortality in early reperfusion group was lower (8.1%; CI, 0.02 to 0.168) than that in the central repair group (16.2%; CI, 0.115 to 0.216). The pooled long-term mortality was 7.9% in the early reperfusion cohort and 17.4% the central repair-first cohort, without a statistically significant heterogeneity (I [2] = 51.271%; *p* = 0.056). The mean time of symptom-onset-to-the-operation-room in all the reports was 8.87 ± 12.3 h.

**Conclusion:**

This meta-analysis suggested that early reperfusion-first may achieved better outcomes compared to central repair-first in ATAAD patients complicated with CM to some extent. Early operation and early restoration of cerebral perfusion may reduce the occurrence of some neurological complications.

**Trial registration:**

: The meta-analysis was registered in the International Prospective Register of Systematic Reviews database (No. CRD CRD42023475629) on Nov. 8th, 2023.

## Background

Cerebral malperfusion (CM) is a common comorbidity in acute type A aortic dissection, with an approximate incidence of 15.9%, an increasing in-hospital mortality rate (20.1%) [1] and poor neurological prognosis[2]. Following antegrade and/or retrograde cerebral perfusion, 35.7% (71/199) of patients showed no improvement or further deterioration of the nervous system. However, current therapeutic strategies remain controversial. Some researchers suggested early reperfusion (expose the carotid artery and immediate carotid reperfusion) followed by delayed open central repair (median thoracotomy with total arch replacement complemented by selective cerebral perfusion), considering that prolonged ischemia could cause clinical irreversible dysfunction [3], however researchers in most cardiac centers are still prone to central aortic repair-first.

This systematic review and meta-analysis aimed to analyze the status of the surgical strategy for ATAAD patients complicated with CM, and compare the difference of therapeutic effectiveness between the central repair-first strategy and the early reperfusion-first strategy according to the clinical outcomes sourced from relevant studies.

## Methods

The meta-analysis was performed following the Preferred Reporting Items for Systematic Reviews and Meta-analyses (PRISMA) guidelines and was registered in the International Prospective Register of Systematic Reviews database (No. CRD CRD42023475629).

### Search strategy

A systematic literature review (covering studies published between 1984 and 2023) was conducted in major databases, including PubMed, Embase, and the Cochrane Library. To identify all relevant studies in these databases, following keywords and medical subject heading terms were applied: Ascending aorta dissection, Type A or Stanford A or DeBakey Type I or DeBakey Type II aorta dissection, cerebral malperfusion, brain malperfusion, unconsciousness or coma, stroke, neurological complications. To enhance detection, relevant studies were browsed with full reference checks. Full texts were screened by the inclusion criteria in sequence as listed below. Relevant articles were reviewed and selected for inclusion by Co-authors (K. H. and XL. Q.). Differences were addressed by discussing to reach a consensus.

### Inclusion criteria

Studies were considered eligible when meet following criteria (1) patients were diagnosed of ATAAD complicated with CM by preoperative vascular contrast-enhanced CT (2), the baseline, perioperative period data were reported in the study (3) outcome indicators in the study included at least one of the following types: in-hospital or short-term mortality (30 days post operative), long-term survival rate, long-term morality rate.

### Exclusion criteria

Separately, studies meet either one of the following conditions were excluded from this meta-analysis: [1] reviews, case reports, conference abstracts, and letters; [2] studies with data reported ambiguously [3]. CM was diagnosed during or after surgery. All studies were selected by two independent investigators (K. H. and XL. Q.), and any disagreements were resolved by discussing to reach a consensus.

### Quality assessment

Each included study was evaluated and scored by two authors (Kang He and Xiaoli Qin) to estimate its quality according to the Methodological index for non-randomized studies (Minors) checklist [4]. All differences were discussed by consensus with third-party recommendations. After scoring, we chose to include studies with a score greater than 8.

### Data extraction

Data of interest were extracted from full texts, tables, and figures, including the first author, time of the study, study design, baseline demographics, interventions, follow-up, and outcomes, which was proceeded by two researchers (K. H. and XL. Q.) independently, and disagreements between them were identified and resolved by discussing to reach a consensus. Baseline demographics covered age, gender, comorbidity, preoperative neurological presentation, the time from onset of symptoms to operation, surgery procedure, the mortality rate, the neurological outcomes and other complication. Outcomes of interest were defined as follows: early mortality was defined as in-hospital mortality or within 30-day mortality after surgery due to any reason, long-term survival was the time from discharge to death.

### Statistical analysis

Statistical analysis was conducted by RevMan (version 5.4) and Stata version 15.0. All P-values were two-sided, and *P* < 0.05 was considered as statistically significant. Forest plots were generated to display the pooled results. A random-effect model with Mantel–Haenszel weighting was used to estimate the overall risk ratio (RR) and 95% confidence interval (CI) values for variables with dichotomous outcomes, including early mortality. Heterogeneity was tested by the chi-squared test and quantified by the I² statistic. Substantial heterogeneity was confirmed when *P* < 0.05 or I [2] > 50%. Dichotomous variables were displayed as counts and percentages, while continuous data were presented as mean ± standard deviation values in the case of normally distributed data. Subgroup analysis was performed according to different outcomes.

## Result

### Study characteristics

**Fig. 1:**
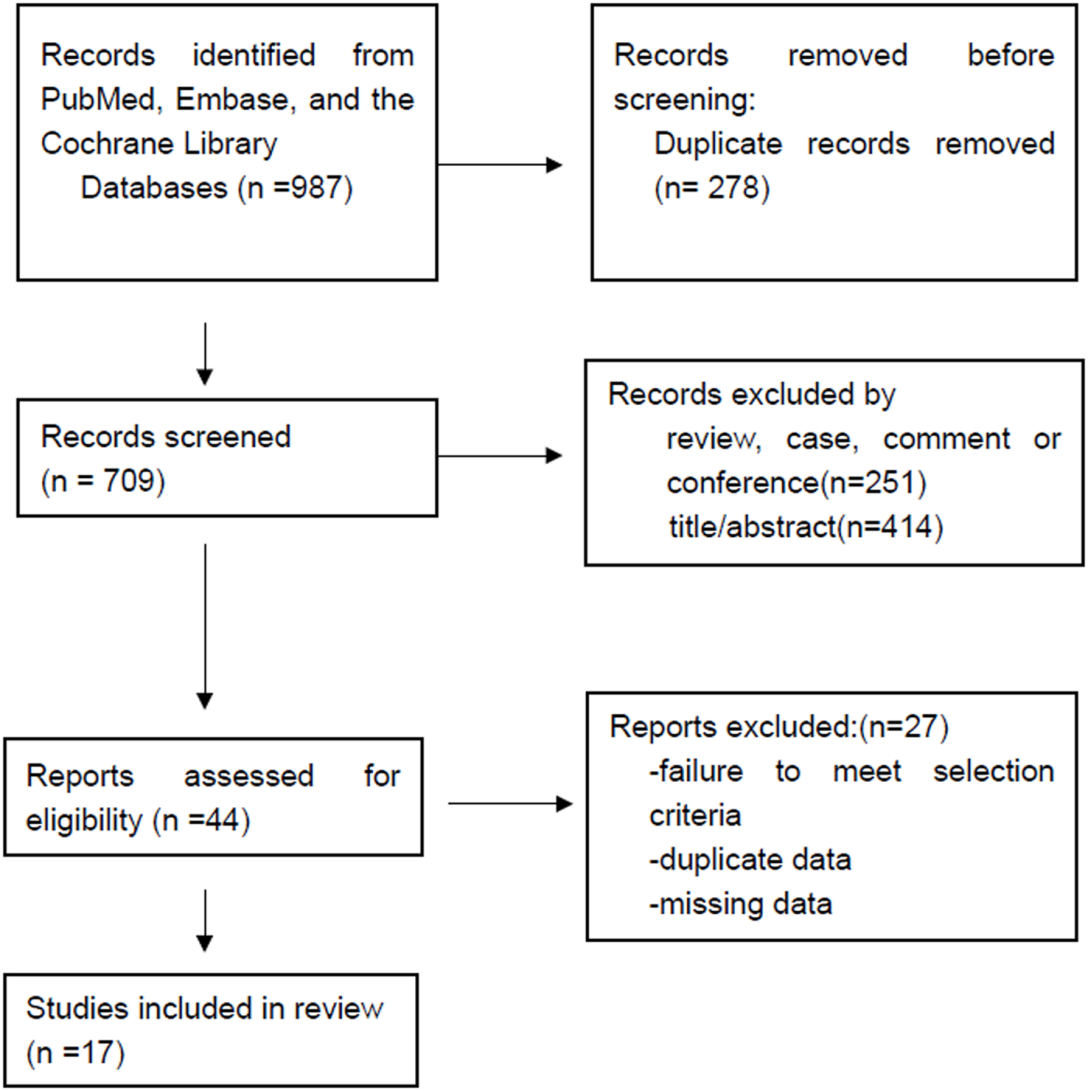
Flow diagram of literature search, selection and exclusion of articles used for the review

Based on the search strategy, a total of 987 relevant publications was screened out from Pubmed, Cochrane and Embase, out of which 17 studies^5–22^, focusing on the treatment of ATAAD complicated with CM, ranging from 1984 to 2023, finally met the inclusion criteria. All included reports were retrospective studies, no relevant randomized controlled trial was identified after researching the databases. Fig. 1 showed the selection process according to the PRISMA guidelines. The included study characteristics were presented in Table 1.

**Table 1:** Study characteristics of the article included in the systematic review and meta-analysis. CM: Cerebral malperfusion

11	Article types	Year	Country	Study period	CM, *n*	Follow-up	Ends points	Group focused	Minors Score
Sun	Retrospective analysis	2023	China	2021–2022	28	16.5(11.5–20.5) months	30-days mortality, long-term survival rate	early reperfusion	12
Xue	Retrospective analysis	2021	China	2011–2019	131	NA	30-days mortality rate	central repair, CM VS.no-CM	10
Sultan	Retrospective analysis	2021	IRAD	2010–2017	362	NA	Hospital mortality, long-term survival rate	CM VS.no-CM	10
Sugiyama	Retrospective analysis	2021	Japan	2015–2020	19	NA	Hospital mortality, long-term mortality rate	central repair and early reperfusion	9
Gomibuchi	Retrospective analysis	2019	Japan	2009–2017	42	NA	Hospital mortality, 30-days mortality rate	central repair and early reperfusion	11
Fukuhara	Retrospective analysis	2021	Japan	1996–2019	80	NA	Hospital mortality rate	NA	8
Shimura	Retrospective analysis	2020	Japan	2007–2017	16	101 ± 7 months	Hospital mortality, long-term mortality rate	central repair	12
Sasaki	Retrospective analysis	2020	Japan	2012–2017	9	24 months	Hospital mortality, long-term mortality rate	early reperfusion	12
Keribich	Retrospective analysis	2019	Germany	2002–2017	150	3.4(1.8–4.6) years	Hospital mortality, 30-days mortality rate	central repair	11
Dumfarth	Retrospective analysis	2020	Austria	2000–2017	50	39.7 ± 51 months	30-days mortality, long-term survival rate	central repair	10
Okita	Retrospective analysis	2017	Japan	1999–2017	9	NA	Long-term survival rate	early reperfusion	8
Chiu	Retrospective analysis	2018	American	2005–2015	50	1.3 yeas	Hospital mortality, long-term survival rate	CM VS.no-CM	11
Luehr	Retrospective analysis	2015	Germany	2005–2013	23	15.2 months	Hospital mortality, long-term mortality rate	early reperfusion	12
Tsukube	Retrospective analysis	2014	Japan	2003–2013	24	55.6 ± 45.7 months	Hospital mortality, long-term survival rate	central repair	10
Niclauss	Retrospective analysis	2013	Switerland	1999–2010	17	30 days	Hospital mortality rate	CM VS.no-CM	9
Morimoto	Retrospective analysis	2011	Japan	1999–2008	41	4.8 years	Hospital mortality, long-term survival rate	central repair	12
Estrera	Retrospective analysis	2006	Amercian	1999–2005	16	18.5 months	Hospital mortality, long-term survival rate	central repair	9

9 out of 17 studies focused on the central repair strategy, while, 6 out of 17 studies considered using early reperfusion strategies to treat cerebral malperfusion, and 2 out of 17 studies involved both strategies. In addition, 4 studies reported the comparison of management and outcomes between patients with AD and CM and patients without CM. It’s worth noting that nearly 60% of the literature outcomes came from different medical centers in Japan.

### Clinical characteristics

The relative clinical characteristics of patients who were treated by central repair or early reperfusion strategy during surgery were presented in Table 2. The pooled effect sizes of all characteristics were calculated using RevMan version 5.4. In the central repair group and early reperfusion group, the mean cardiopulmonary bypass (CPB) time was 220.7 ± 94.5 and 220.3 ± 68.1 min (MD, 0.4; 95% CI, − 18.42 to 19.22; *p* = 0.97), the aortic cross-clamp (Acc) time was 141.3 ± 76.5 and 117.3 ± 48.5 min (MD, 24; 95% CI, 15.48 to 32.52; *p* < 0.01), respectively. The hypothermic cardiac arrest (HCA) time was 35.1 ± 18.2 min in the central repair group and 39.4 ± 15.7 min in early reperfusion group (MD, -4.30; 95% CI, -16.07 to 7.47, *p* = 0.47). Antegrade cerebral perfusion (ACP) was applied to protect brain for all the patients in early reperfusion group compared with 68% cases in central repair group selected retrograde cerebral perfusion (RCP) (MD, 0.68, *p* < 0.01).

**Table 2:** Clinical characteristics of participants in central repair group and early reperfusion group.DM: diabetes mellitus; COPD: chronic obstructive pulmonary disease; RCCA: right carotid common artery; LCCA: left carotid common artery; BCCA: bilateral common carotid artery; RR: risk ratio; CPB: cardiopulmonary bypass; Acc: aortic cross-clamp; HCA: hypothermic cardiac arrest; ACP: antegrade cerebral perfusion; RCP: retrograde cerebral perfusion

Variable	Central repair, *n* (%)	Early reperfusion, *n* (%)	RR, MD	*P*
**Number of patients**	472	86		
**Mean age, years**	61.1 ± 17.3	60.9 ± 16.4	0.20(-3.73, 4.13)	0.92
**Sex, male**	189/322	54/86	0.93(0.78, 1.13)	0.48
**Hypertension**	187/270	50/68	0.94(0.80, 1.11)	0.47
**DM**	8/181	5/23	0.20(0.07, 0.57)	0.002
**COPD**	3/143	1/23	0.48(0.05, 4.44)	0.52
**Coronary artery disease**	16/234	3/28	0.64(0.20, 2.05)	0.45
**Renal insufficiency**	3/143	12/58	0.10(0.03, 0.35)	<0.01
**Aortic valve insufficiency**	6/44	15/50	0.45(0.19, 1.07)	0.07
**Organ malperfusion**				
Coronary	36/341	10/61	0.64(0.34, 1.23)	0.18
Mesenteric	30/293	0/28	6.02(0.38, 95.86)	0.20
Limb	64/341	6/68	2.13(0.96, 4.71)	0.06
Kidney	24/182	2/38	2.51(0.62, 10.16)	0.2
**Cardiac tamponade**	78/391	4/40	1.99(0.77, 5.16)	0.15
**Preoperative presentation**				
Coma	79/436	13/60	0.84(0.50, 1.41)	0.50
Hemiplegia	120/257	14/69	2.30(1.42, 3.74)	< 0.001
Neurologic deficit	15/44	14/45	1.10(0.60, 1.99)	0.76
Syncope	15/150	7/23	0.33(0.15, 0.72)	0.005
Hemianopsia	30/207	1/23	3.33(0.48, 23.32)	0.23
Shock	5/44	9/68	0.86(0.31, 2.39)	0.77
**Involved artery**				
RCCA	142/248	30/51	0.97(0.76, 1.25)	0.83
LCCA	91/248	12/61	1.87(1.09, 3.18)	0.02
BCCA	9/32	5/70	3.94(1.43, 10.81)	<0.01
Innominate artery	1/16	21/37	0.11(0.02, 0.75)	0.02
**Time to surgery, h**	8.2 ± 15.3	7.8 ± 5.4	0.4(-1.6, 2.40)	0.7
**Surgery procedure**				
Hemiarch	87/396	18/67	0.82(0.53, 1.27)	0.37
Partial arch	2/53	3/35	0.44(0.08, 2.50)	0.35
Total arch	128/396	46/67	0.47(0.38, 0.58)	<0.01
Ascending replacement	43/61	8/51	4.49(2.33, 8.67)	<0.01
Bentall	55/303	26/51	0.36(0.25, 0.51)	<0.01
Valve sparing	1/153	24/51	0.01(0, 0.1)	<0.01
AVR	1/12	11/23	0.17(0.03, 1.19)	0.08
CABG	3/12	10/51	1.27(0.41, 3.93)	0.67
Elephant trunk	39/108	32/51	0.58(0.41, 0.80)	0.001
**Arterial cannulation**				
Axillary artery	101/344	15/51	1.00(0.63, 1.57)	0.99
Femoral artery	127/344	35/51	0.54(0.43, 0.68)	<0.01
Carotid artery	4/150	27/32	0.03(0.01, 0.08)	<0.01
Aortic	56/150	1/53	19.79(2.81, 139.41)	0.003
**Cerebral perfusion**				
ACP	284/420	86/86	0.68(0.63, 0.73)	<0.01
RCP	110/420	0/86	45.67(2.87, 727.71)	0.007
**Lowest temperature, °C**	21.7 ± 3.3	25.3 ± 2.4	-3.60(-4.32, -2.88)	<0.01
**CPB, min**	220.7 ± 94.5	220.3 ± 68.1	0.4(-18.42, 19.22)	0.97
**Acc, min**	141.3 ± 76.5	117.3 ± 48.5	24(15.48, 32.52)	<0.01
**HCA, min**	35.1 ± 18.2	39.4 ± 15.7	-4.30(-16.07, 7.47)	0.47

### In-hospital/30‐day mortality

**Fig. 2:**
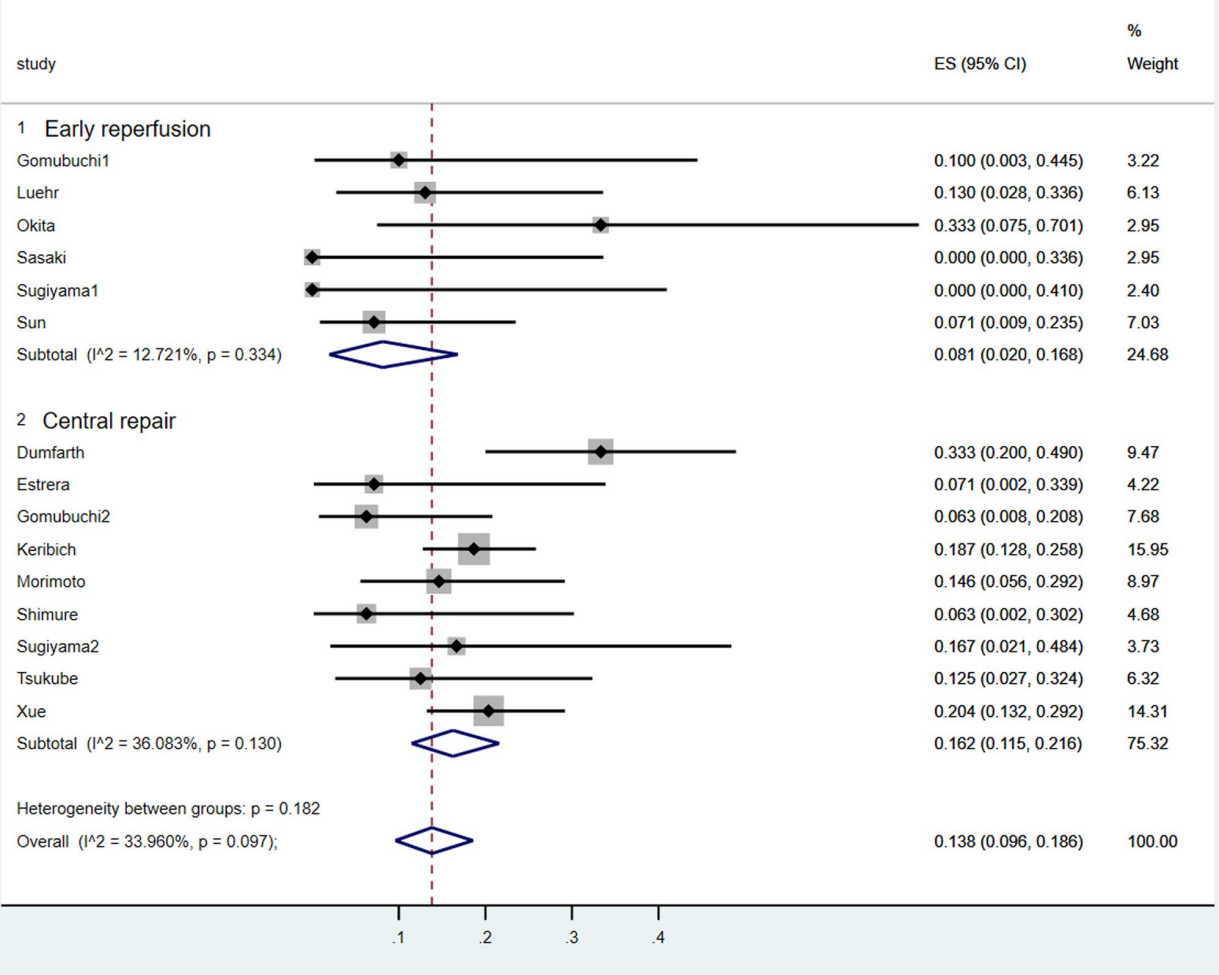
Forest plots of the meta-analysis depicting early mortality of patients in central repair group and early reperfusion group, respectively

**Fig. 3:**
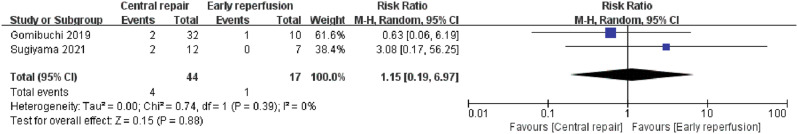
Forest plots of the meta-analysis depicting early mortality of patients with CM in two studies

**Fig. 4:**
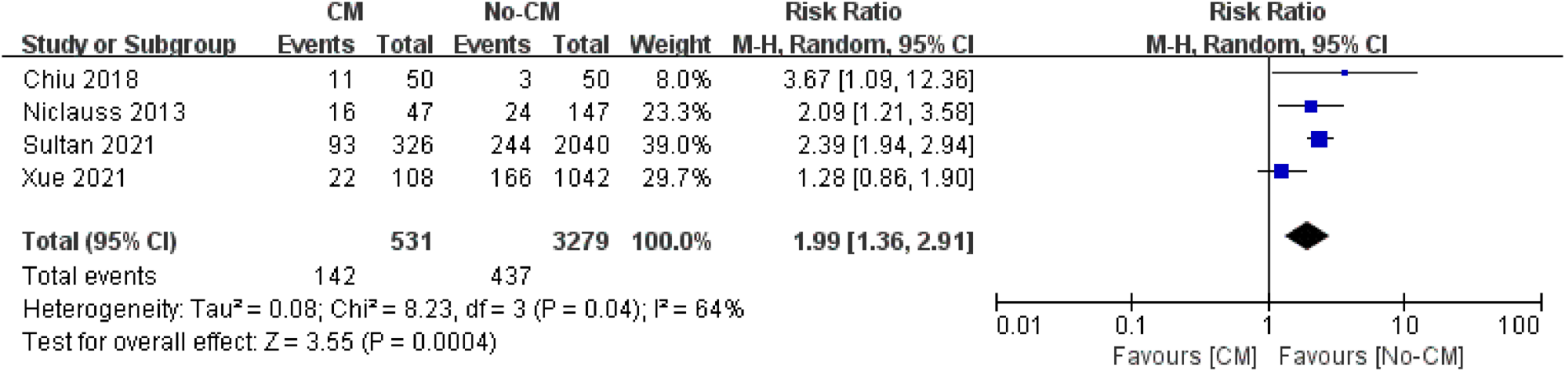
Forest plots of the meta-analysis depicting early mortality of patients with /without CM

This analysis included cases collected across a large time span period which were characterized with different kind of symptoms, comorbidities and surgery techniques. A total of 13 studies focusing on the influence of central repair and early reperfusion strategy were included in this analysis with a cumulative cohort of 86 patients in early reperfusion group and 472 patients in central group, based on which pooled in-hospital/30‐day mortality were estimated. The pooled early mortality in early reperfusion group was lower than that in the central repair group, and the difference between two groups was statistically significant (8.1% vs.16.2%) (Fig. 2). In addition, according to the forest plot of the meta-analysis which presented the difference in perioperative results and outcomes between the two treatment strategies, although no statistical significance was found, the forest plots indicated a lower early mortality of early reperfusion (*P* = 0.88; RR, 1.15; 95% CI, 0.19 to 6.97) (Fig. 3). Patients without CM were demonstrated to have a significantly lower in‐hospital/30‐day mortality when compared to patients with CM, with a pooled RR of 1.99 (*p* < 0.01, 95% CI: 1.36–2.91) (Fig. 4). A random‐effect model was used in the quantitative synthesis above, and the heterogeneity between two groups was found to be statistically significant (*p* = 0.04 and I [2] = 64%).

### Long-term mortality and survival rate

**Fig. 5:**
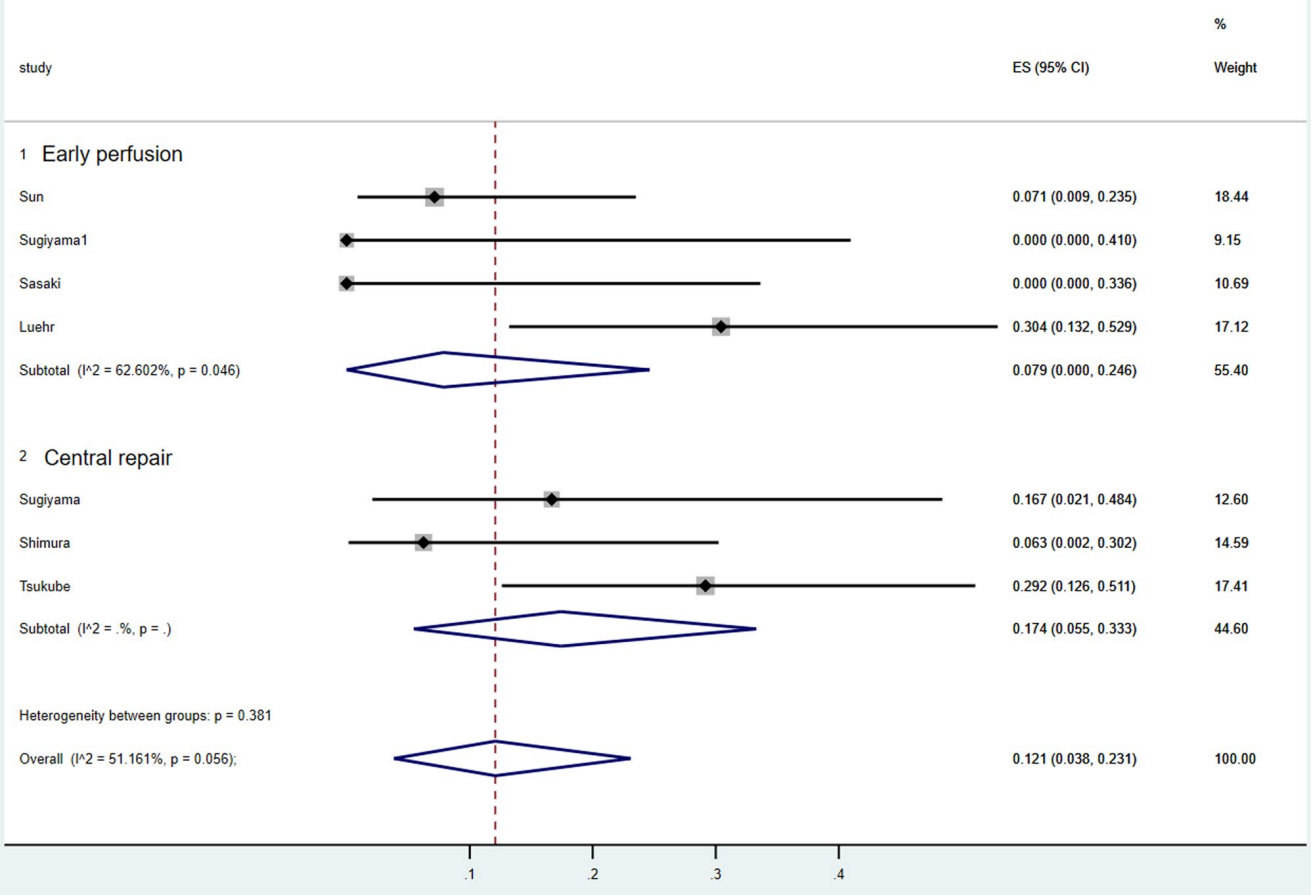
Forest plots of the meta-analysis depicting long-term mortality of patients in central repair group and early reperfusion group, respectively

As shown in Fig. 5, the pooled long-term mortality was 7.9% in the early reperfusion cohort and 17.4% the central repair-first cohort, without a statistically significant heterogeneity (I [2] = 51.271%; *p* = 0.056). Among these studies, the five-year survival was reported in four studies (75.2%±12.5%, 65%±8%, 60.3%, 58%), and the 10-year survival was reported to be 60.5 ± 23.4% and 59% ± 9% respectively in two studies. Sun et al. reported the highest long-term survival rate (92.9%, 26 / 28) among all the studies.

### Time to surgery

The mean time that lapsed between the onset of symptoms and surgery was 8.87 ± 12.3 h. The mean symptom-onset-to-operation-room time was 7.7 ± 5.4 h in the early reperfusion group and 9.3 ± 13.5 h in the central repair group (-1.6; 95% CI, -3.33 to 0.13). There was no significant difference in the time intervals between the 2 groups (*P* = 0.07).

### Perioperative neurological presentation

With regard to preoperative presentation, the patients in early reperfusion group appeared to be more likely to suffer from hemiplegia (MD, 2.30; 95% CI, 1.42 to 3,74, *p* < 0.01) and syncope (MD, 0.33; 95% CI, 0.15 to 0.72, *p* = 0.001, compared with the patients in central repair group). In the documented preoperative neurological presentation, coma (*n* = 290) was the most frequently occurred event. The rare presentation of hemianopsia was detected in 31 patients. In seven studies, postoperative CVA was reported including 180 (24.2%) patients. Five case series documented 44 post-coma patients (10.4%). Temporary and transient postoperative neurological deficits was 149 (23.1%), hemiplegia was 42 (7.2%).

### Postoperative neurological complications

With regard to preoperative presentation, cerebral complications in early reperfusion were 34.8% (8/23) vs. 34.3% (118/344) in central repair group. 33.3% and 7.5% patients had coma in early reperfusion and central repair group, respectively. 44.2% patients had nervous system dysfunction in central repair group, whereas in the early reperfusion group it was 26.4%.

## Discussion

Cerebral malperfusion is a common comorbidity in acute type A aortic dissection, which could cause a high mortality and a poor neurological prognosis. According to previously reported data from the International Registry of Acute Aortic Dissection, 6% of patients with ATAAD present with CM before surgery [23, 24]. ATAAD patients with CM presented obviously different baseline characteristics and manifestations, and had worse outcome, compared with ATAAD patients without CM. In our meta-analysis the analysis and comparison of managements and outcomes was conducted between ATAAD patients with CM and ATAAD patients without CM based on 5 studies, which included a total of 3873 cases (547 CM and 3326 no-CM). Regarding the early mortality, data from this meta-analysis indicated that no-CM patients had significant better outcome than CM patients.

ATAAD with CM has an unfavorable prognosis and high mortality, and early treatment may lead to better outcomes. However, current therapeutic strategies remain controversial. Except for assessing patients’ condition, it is critical to choose an appropriate therapeutic strategy between central repair and branch reperfusion. At present, most of medical centers still suggest that central repair composed of entry closure and vascular remodeling was the first procedure to improve patients’ survival rate and neurological outcomes. However, some patients who had serious preoperative neurological presentation would suffer from irretrievable defects after central repair [25, 26].

The pathophysiology of cerebral malperfusion in ATAAD patients is largely due to a true lumen compressed by a false lumen [15], resulting in circulatory collapse, acute hypoxia, and thromboembolism originated from the false lumen. Meanwhile, postoperative neurological deficiency may be caused by disturbed blood flow in stenotic or occluded carotid artery [9].

Axillary artery and femoral artery are still the most used cannulation sites for surgery strategy. Cannulation of the axillary artery or ascending aorta may not restore the true lumen, instead this may cause cerebral hypoperfusion because of static malperfusion [26]. Selective anterograde cerebral perfusion via axillary artery and retrograde cerebral perfusion via superior and inferior vena cava might work in limited cases to restore brain blood, but not be able to restore true-lumen-caused systemic hypoperfusion [27, 28]. For the prevention of irreversible brain injury, preoperative assessment and intraoperative management are important. It’s critical to control the risk of increased cerebral ischemia due to central repair and metabolic derangement, or aortic rupture due to reperfusion first. Nonetheless, the optimal intraoperative strategy remains controversial [27].

Restoration of cerebral blood flow via conventional central aortic repair with selective cerebral perfusion may be very difficult if severe stenosis or occlusion of the common carotid arteries is present. According to postoperative CT results, cerebral infarction may be caused by thromboembolism and carotid artery occlusion before surgery [29]. Removing thrombi and recovering the carotid artery blood flow could reduce the duration of brain ischemia and achieve better prognosis [15]. 2020 JCS Guideline suggested to adopt extra-anatomical aorta-carotid artery bypass surgery for minimizing the cerebral ischemia time before performing central repair. Central repair for carotid artery malperfusion with acute dissection should be considered to improve carotid artery blood flow in the hyperacute phase [30].

Early reperfusion strategy had been introduced in several studies. Sun et al. [5] chose to anastomose prosthetic graft above the occlusion level of the carotid artery, and the ends of the prosthetic grafts were anastomosed to the extracorporeal circulation pump tube. Sugiyama [8] et al. have reported seven cases using direct perfusion to the carotid artery in the operating room by the selective cerebral perfusion circuit, and no death during hospitalization or worsened neurological outcome was reported. Stenosed right or left carotid artery was exposed at the beginning of the surgery. The vessel was then cannulated directly with a proximal clamp and cut-down technique above the occlusion level. When the common carotid artery was transected, all observable thrombi were moved from the false lumen. Gomibuchi [9] transected aortic arch, or transected the carotid artery after common carotid artery was exposed through an oblique neck incision anterior to the sternocleidomastoid muscle, then a 12-Fr selective cerebral perfusion cannula was applied and directly inserted into the true lumen of the carotid artery. Three reasons why early reperfusion could avoid the occurrence of postoperative neurological comorbidity was proposed by the researchers: (1) Early reperfusion could reduce the duration of brain ischemia; (2) It prevented cerebral hemodynamic instability during surgery; (3) The anatomy of the carotid artery helped clear away the thromboembolism in the false lumen. Sasaki et al. [12] considered that definitive blood flow restoration under direct vision with hypothermic circulatory arrest could avoid creating a new intimal tear and embolism. The exposed carotid artery, which had been transected and sutured with prosthetic graft, was utilized to recover brain blood flow after hypothermic circulatory arrest. Although catheterization to the carotid artery is quick and can reduce ischemic time [31], it can potentially create a new intimal tear and embolism in fragile vessels. A total of 9 patients’ outcomes were reported, including 100% of overall survival and significantly improved hemiplegia and hemiparesis. Okita et al. [15] reported a simple bypass circuit between the common femoral artery which was chosen for arterial drainage and right common carotid artery which was inserted directly by arterial cannula. Luehr [17] performed aorto-carotid bypass and bilateral selective cerebral perfusion: surgery was performed via combining right common carotid artery prosthetic graft with direct left common carotid artery cannulation in 23 patients. All the outcomes of case series which used revascularization and early cerebral reperfusion strategy were concluded in our meta-analysis, and the pooled early mortality was calculated to be 8.1%, which was lower than that in central repair group (16.2%). And the pooled long-term mortality was 7.9% in the early reperfusion cohort and 17.4% the central repair-first cohort.

In most of reports regarding early reperfusion strategy, surgeons tended to expose the carotid artery before the start of open central repair and recover cerebral perfusion. Anastomosing prosthetic graft to reconstruct the anatomical structure of occlusion carotid artery was the most common choice for surgeon, of course some patients had to be insert transcatheter carotid artery perfusion cannula in emergency room because patients were in critical condition. Zhan et al. [32] and Inoue et al. [33] suggested that artery anatomical structure changes may be the key to improve preoperative neurological symptoms and reduce the occurrence of postoperative neurological complications. Notably, supra-aortic branch vessels are frequently involved in ATAAD, meanwhile, dissection could result in unilateral or bilateral carotid artery stenosis, and thrombosis could cause carotid artery occlusion, as a result, putting aside the preoperative neurological symptoms, carotid blood flow disorders or the occlusion of supra-aortic branch vessels could lead to poor postoperative neurological outcomes [34]–[36]. Before central repair, prosthetic graft and perfusion cannula for reconstructing carotid artery blood flow, along with selection cerebral perfusion in the surgery can solve dynamic and static cerebral malperfusion. Early perfusion can rapidly relieve organ ischemia, reduce organ ischemia time, meanwhile, maintain the cerebral hemodynamic stability during surgery, reduce the high incidence of postoperative neurological complications in patients with ATAAD and cerebral malperfusion. However, prolonged treatment of carotid artery may increase the risk of aortic rupture. Early carotid artery perfusion strategy is not actively adopted in lots of hospitals, in stead, selective cerebral perfusion via axillary artery or right common carotid artery after transecting aortic arch is more frequently adopted.

Many patients were referred from primary hospital to local cardiac medical center, so it is hard to precisely estimate the time interval between the symptom presentation and hospitalization. Previous study reported that early surgery might lead to better clinical outcomes, but the limitation of time before surgery haven’t been defined clearly. Estrera et al. [21] reported 14 aortic dissection patients with preoperative stroke, and 80% of them who underwent surgical repair within 10 h achieved improvements in neurologic status, while those beyond 10 h did not. Tsukube and colleagues [18] have previously reported that operations beyond 5 h might be associated with worse outcomes. When the time was less than 5 h, hospital mortality was 14% and 86% of patients achieved full recovery of consciousness. The mean symptom-onset-to-operation-room time in all the reports included in our meta-analysis was 8.87 ± 12.3 h. The mean symptom-onset-to-operation-room time was 7.7 ± 5.4 h in the early reperfusion group and 9.3 ± 13.5 h in the central repair group. So early operation may improve their early and long-term outcomes to a certain extent.

### Limitations

There are several limitations in our current meta-analysis. Firstly, since only 2 studies had data from both two strategies used in the same center, all other studies were single-arm studies, there is a certain risk of bias. Secondly, the lack of complete data in some studies impeded us from analyzing the relevant results, such as long-term survival rate, which might cause potential deviation. Most of the included studies were single-arm intervention researches, with only two included the comparison of early reperfusion and central repair strategies. On the basis of current cases, it is insufficient to draw an accurate conclusion of the differences in clinical outcomes and prognosis between the two strategies. Thirdly, surgical strategy was sometimes chosen by the surgeons, and different centers may have some differences of surgical methods, which may lead to heterogeneity. Fourth, time from onset of symptoms to operating room is a challenging data point, due to the fact that many of these patients are transferred from multiple hospitals.

## Conclusion

In conclusion, ATAAD patients with CM have a significantly higher in-hospital/30‐day mortality. The strategy of early reperfusion can achieve a lower early mortality than aortic central repair, possibly making it the preferred treatment of choice to some extent. Early operation and early restoration of cerebral perfusion may reduce the occurrence of some postoperative neurological complications in patients with preoperative cerebral malperfusion. At this point, a meta-analysis based on more studies is necessary to re-verify our current conclusions and help with better clinical decision in the near future.

## Data Availability

Data is provided within the manuscript.

## Abbreviations

CM: cerebral malperfusion
ATAAD: acute type A aortic dissection
DM: diabetes mellitus
COPD: chronic obstructive pulmonary disease
RCCA: right carotid common artery
LCCA: left carotid common artery
BCCA: bilateral common carotid artery
RR: risk ratio
CPB: cardiopulmonary bypass
Acc: aortic cross-clamp
HCA: hypothermic cardiac arrest
ACP: antegrade cerebral perfusion
RCP: retrograde cerebral perfusion
